# Dermatological Ultrasound of Less Frequent Dermatological Vascular Anomalies: A Retrospective Study and Literature Review From a Reference Center in Bogotá, Colombia

**DOI:** 10.7759/cureus.70400

**Published:** 2024-09-28

**Authors:** Claudia Gonzalez, Valeria Duque-Clavijo, Juliana Cantor, Hernán Emilio Duque-Romero

**Affiliations:** 1 Radiology, Rosario University, Bogota, COL; 2 Medicine, School of Medicine, Andes University, Bogota, COL; 3 Dermatology, UDL Dermatological Group, Pereira, COL

**Keywords:** angiokeratoma, cherry angiomas, cutaneous angiosarcoma, dermatological ultrasound, extradigital glomus tumors, pyogenic granuloma, verrucous venous malformation

## Abstract

Vascular anomalies (VA) are classified as either vascular tumors or vascular malformations. Vascular tumors are characterized by the neoplastic proliferation of endothelial cells, whereas vascular malformations result from defects in the pathways that regulate the development of vascular channels during embryogenesis. Dermatological ultrasound has emerged as a critical diagnostic tool for distinguishing VA from other conditions and differentiating among various types of VA by providing detailed imaging characteristics.

In this study, we present cases of less common VA identified and characterized using dermatological ultrasound to illustrate the utility of this modality in clinical practice. Patients were randomly selected from clinical records (February 2021 to February 2024) with dermatological VA confirmed by histology, spanning ages 10 to 72 years. Inclusion required complete clinical records and ultrasound reports. The sample represents six different VA and illustrates a spectrum of complications observed in clinical practice.

The selected cases include five vascular tumors: pyogenic granuloma, cutaneous angiosarcoma, extradigital glomus tumors, cherry angiomas, and angiokeratoma, and one vascular malformation: verrucous venous malformation. Despite their varied clinical presentations, dermatological ultrasound demonstrated distinct features for each type of anomaly, proving to be a valuable tool in the multidisciplinary management of cutaneous VA. This imaging modality enables precise evaluation and guides effective treatment strategies.

## Introduction

Vascular anomalies (VA) have been classified by the International Society for the Study of VA (ISSVA) into vascular tumors and vascular malformations. Vascular tumors encompass a diverse group of neoplasms arising from blood or lymphatic vessels in the skin and subcutaneous tissues and are categorized into three primary types: benign, locally aggressive/borderline, and malignant. In contrast, defects in pathways that regulate the development of vascular channels during embryogenesis lead to vascular structural anomalies. These anomalies occur without the neoplastic proliferation of endothelial cells characteristic of vascular tumors. Vascular malformations are further sub-classified into four types: simple, combined, major named vessels, and those associated with other anomalies [1,2].

Dermatological ultrasound is an invaluable tool for clinicians, enabling the differentiation between various VA and other conditions, as well as distinguishing among different types of VA themselves [3-6]. This retrospective study reviews dermatological ultrasound reports from a reference imaging center in Bogotá, COL, over a three-year period. It focuses on diagnosing uncommon dermatological VA, presenting six cases to provide dermatologists with a framework for differentiating these pathologies and enhancing patient care.

## Materials and methods

Study design and setting

This retrospective study was conducted at a reference imaging center in Bogotá, COL, over three years. The primary aim was to illustrate the utility of dermatological ultrasound in identifying and characterizing dermatological VA.

Patient selection

Patients were randomly selected from clinical records spanning from February 2021 to February 2024, with the inclusion criteria being dermatological VA confirmed through histological studies and having complete clinical records and dermatological ultrasound reports. The age range was extended from 10 to 72 years to encompass the occurrence of these anomalies across various age groups. To ensure a representative sample of the six VA studied, patients meeting these criteria were randomly selected. The cases included five vascular tumors, namely pyogenic granuloma (PG), cutaneous angiosarcoma (cAS), extradigital glomus tumors (GTs), cherry angiomas (ChA), and angiokeratoma (Ak), and one vascular malformation (verrucous venous malformation (VVM)).

Ultrasound techniques

Dermatologic ultrasound was performed by an experienced radiologist using a high-frequency linear transducer (>15 MHz), incorporating grayscale imaging and color Doppler duplex analysis, in accordance with established guidelines for dermatologic ultrasound examinations.

Data collection and analysis

Ultrasound reports were systematically reviewed to identify and document the most consistent and characteristic features of each VA. Clinical and ultrasonographic findings were recorded for each case. The data were then summarized to highlight the specific ultrasound features of each type of VA, focusing on distinguishing them from other conditions and one another.

Case presentation

We identified 23 cases, which were categorized as follows: 10 PG, one cAS, five extradigital GTs, four ChA, one Ak, and two VVM. The most pertinent clinical and ultrasonographic findings for each type of VA are presented with a detailing of their distinctive features.

Ethical considerations

The study was conducted in accordance with ethical standards aligned with the Declaration of Helsinki. Informed verbal consent was obtained from all patients prior to the ultrasound examination. Patient data were anonymized to ensure confidentiality, with secure storage and restricted access. Detailed information on consent and data handling practices was provided to uphold the study’s ethical integrity.

Statistical analysis

Descriptive statistics were employed to summarize and present the most relevant clinical and ultrasonographic characteristics of the cases. This included an overview of the features of each type of VA.

## Results

In this study, we selected 23 cases representative of the six pathologies discussed in this article. Table 1 provides detailed information on patient demographics, initial clinical diagnoses, ultrasound findings, and treatments. Table 2 summarizes the most relevant ultrasonographic characteristics of the VA presented. Dermatological ultrasound was used to observe and document the features of various VA, each displaying unique features. Pyogenic granuloma typically appears as a well-defined, exophytic, or polypoid hypoechoic structure with pronounced arterial flow detectable on Doppler. Cutaneous angiosarcoma presents as a nodule or mass with mixed echogenicity and poorly defined borders, showing irregular and tortuous vascular structures on Doppler. Extradigital GTs are characterized by solid, hypoechoic nodules with regular borders and notable arterial and venous flow. Cherry angiomas are identified as thin, superficial lesions confined to the upper dermis with no detectable flow on Doppler. Angiokeratomas display band-like structures with thickening and undulation of the epidermis and minimal vascularity. Finally, verrucous VVM is observed as poorly defined, plaque-like lesions with variable echogenicity and low or absent vascular flow. These observations demonstrate the range of features that dermatological ultrasound can reveal in different vascular anomalies. High-resolution images representing each of the six pathologies are included to provide visual context.

**Table 1:** Demographic and clinical profiles of patients

Age	Gender	Anatomical location	Clinical diagnosis	Ultrasound diagnosis	Histopathological report	Treatment
60	Male	Right temporal region	Telangiectatic granuloma	Telangiectatic granuloma	Telangiectatic granuloma	Surgical excision
46	Female	Right inguinal region	Metastasis	Angiosarcoma	Angiosarcoma	Surgical excision - radiotherapy
23	Male	Right metacarpal region	Synovial cyst	Extradigital glomus tumor	Glomanglioma	Surgical excision
34	Male	Right index finger	Telangiectatic granuloma	Telangiectatic granuloma	Telangiectatic granuloma	Pulsed laser
51	Male	Left hypothenar region	Vascular malformation	Verrucose venous malformation	Verrucose venous malformation	No treatment
6	Male	Left foot sole	Verrucose venous malformation	Cherry angioma	Cherry angioma	Pulsed dye laser
10	Male	Right cheek	Hemangioma	Extradigital glomus Tumor	Glomus tumor	Surgical excision
60	Male	Neck	Angioma	Cherry angioma	Campbell the Morgan spots	No treatment
32	Female	Upper lip	Thrombosed venous lake	Telangiectatic granuloma	Telangiectatic granuloma	Pulsed laser
70	Male	Neck	Cherry angioma	Cherry angioma	Campbell the Morgan spots	Cryocauterization
23	Female	Frontal region	Hemangioma	Extradigital glomus tumor	Glomus tumor	Surgical excision
64	Female	Neck	Cherry angioma	Cherry angioma	Campbell the Morgan spots	No treatment
55	Female	Scalp	Trichilemmal cyst	Telangiectatic granuloma	Lobulillar Capillary hemagioma	Surgical excision
29	Female	Right index finger	Telangiectatic granuloma	Telangiectatic granuloma	Telangiectatic granuloma	Surgical excision
40	Male	Left arm	Telangiectatic granuloma	Extradigital glomus tumor	Glomanglioma	Surgical excision
41	Female	Right pinky finger	Glomic tumor	Telangiectatic granuloma	Lobulillar capillary hemagioma	Surgical excision
10	Male	Left ankle	Verrucose venous malformation	Verrucose venous malformation	Verrucose venous malformation	Surgical excision + laser therapy
28	Female	Nasal dorsum	Foreign granuloma	Telangiectatic granuloma	Telangiectatic granuloma	Surgical excision
46	Female	Right thigh	Verrucose venous malformation	Angiokeratoma	Angiokeratoma	Cryotherapy
31	Female	Right index finger	Glomic tumor	Telangiectatic granuloma	Telangiectatic granuloma	Surgical excision
18	Male	Nasal dorsum	Hemangioma	Telangiectatic granuloma	Telangiectatic granuloma	Surgical excision + laser therapy
25	Male	Left cheek	Glomic tumor	Extradigital glomus tumor	Glomus tumor	Surgical excision
72	Male	Scalp	Trichilemmal cyst	Telangiectatic granuloma	Lobulillar capillary hemagioma	Laser therapy

**Table 2:** Ultrasonographic characteristics of six distinct VA VA: Vascular anomalies, PG: Pyogenic granuloma, cAS: Cutaneous angiosarcoma, GT: Glomus tumors, ChA: Cherry angiomas, Ak: Angiokeratoma, VVM: Verrucous venous malformation

Pathology	B mode		Doppler	
	Appearance	Echogenicity	+/-	Appearance of the vessel
PG	Well-defined, exophytic or polypoid epidermal and dermal hypoechoic structure	Hypoechoic	+++	Normal arterial flow
cAS	Nodule or mass with poorly defined borders	Mix echogenicity predominantly hypoechoic	+++	Disorganized, irregular, tortuous, arterial vessel
GTs	Solid nodule with well-defined, regular borders	Hypoechoic	++	Normal arterial and venous
ChA	Thin and superficial nodule confined to the upper dermis, with well-defined borders, causing upward displacement of the epidermis	Hypoechoic	-	Small capillaries without detected flow
Ak	Band-like epidermal and dermal structures, with thickening, undulation, and irregularities of the epidermis	Hypoechoic	_	Small capillaries without detected flow
VVM	Poorly defined plaque-like lesion with variable degrees of thickening, irregularities, and undulation of the epidermis,	Hypoechoic - hyperechoic in the periphery	-	Small capillaries without detected flow

**Figure 1:**
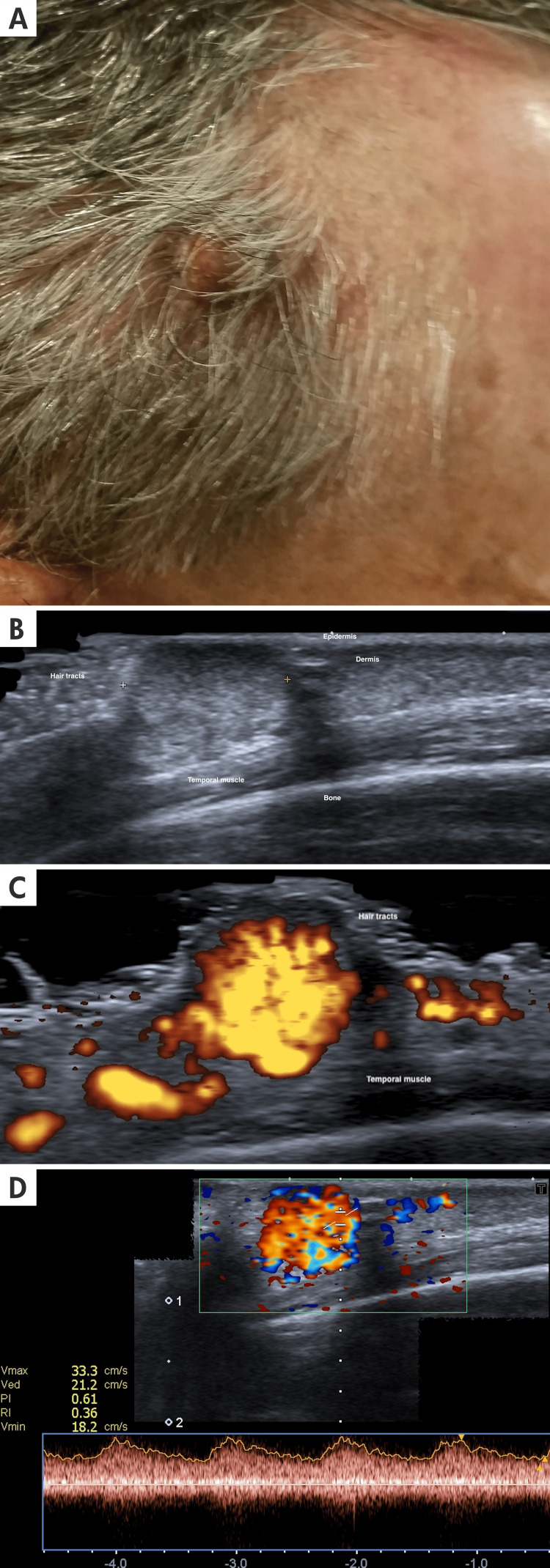
Dermatological ultrasound and clinical imaging of pyogenic granuloma A: A 60-year-old male patient with a rounded, red, tuberous-nodular lesion with an eroded, friable center, well-demarcated, approximately 7 mm in diameter, located in the right temporal region B: The longitudinal ultrasound view and grayscale ultrasound of the temporal region show a hypoechoic, well-defined, exophytic nodule located in the dermo-hypodermal area. The calipers (+) show the diameter of the lesion C: On power Doppler color ultrasound, the lesion has prominent and diffuse hypervascularization. D: The spectral curve shows arterial flow with a systolic peak of 33.3 cm, which is higher than expected for a small vessel in this region.

**Figure 2:**
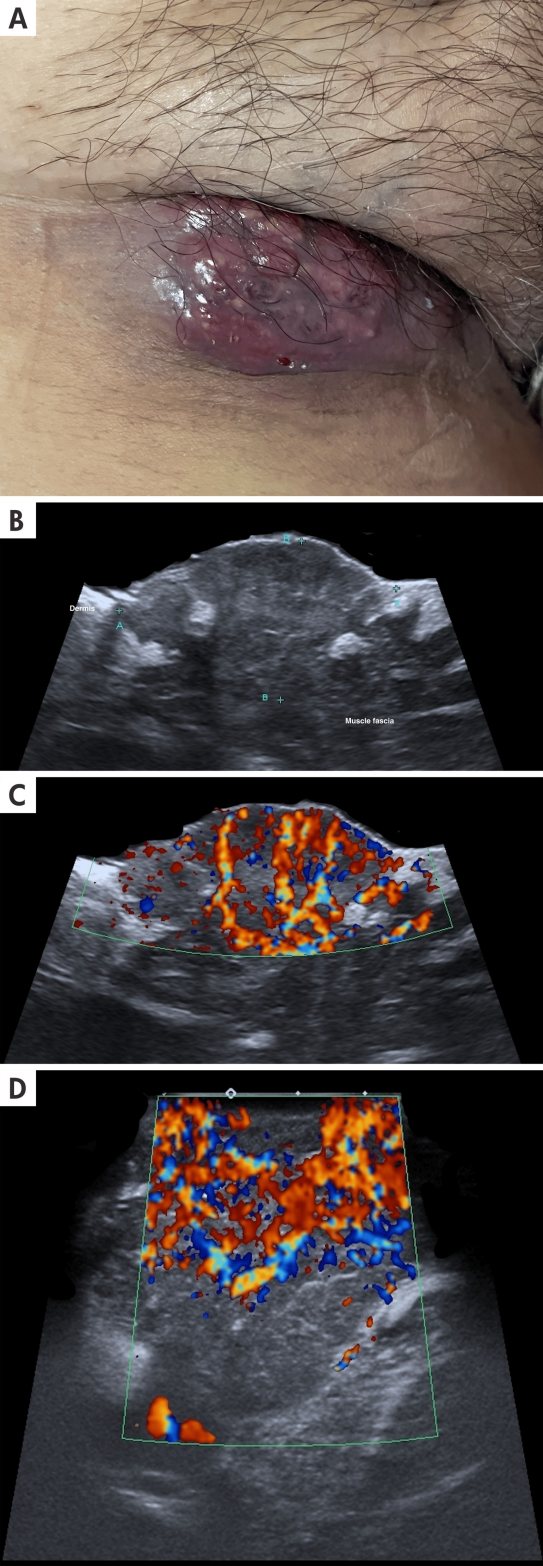
Dermatological ultrasound and clinical imaging of cutaneous angiosarcoma A: A 46-year-old female patient with a lobulated, edematous, red-violet, vascular-appearing tumor lesion, approximately 5 cm x 2 cm in size, with surrounding skin also violet and edematous, located in the right inguinal region. B: The longitudinal ultrasound view and grayscale ultrasound of the inguinal area show a mix of hypoechoic and hyperechoic structures with poorly defined borders. The calipers (A+) show the longitudinal extension, and the calipers (B+) show the anteroposterior borders. C and D: On Doppler, duplex color Doppler color ultrasound, there are multiple, tortuous vessels with irregular borders.

**Figure 3:**
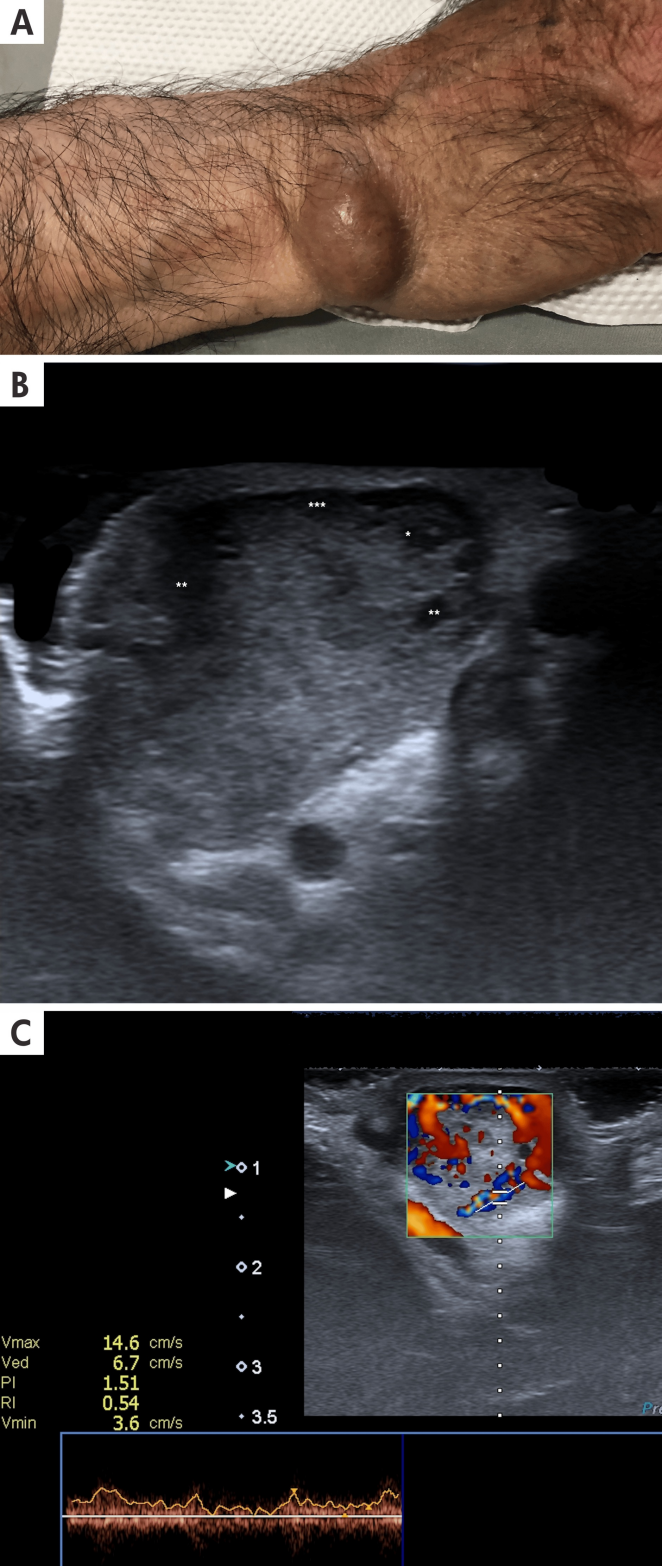
Dermatological ultrasound and clinical imaging of extradigital glomus tumor A: A 23-year-old male patient with a nodular, erythematous-violet, smooth, firm, oval, well-defined, well-demarcated, mobile tumor lesion, approximately 2 cm x 3 cm in size, located in the right metacarpal region. B: The longitudinal ultrasound view and grayscale ultrasound of the wrist region show an exophytic hypoechoic mass with well-defined borders; asterisks (*) show small hypoechoic serpentine capillaries inside the lesion. C: The spectral curve confirms the arterial origin of the vessel within the glomus tumor.

**Figure 4:**
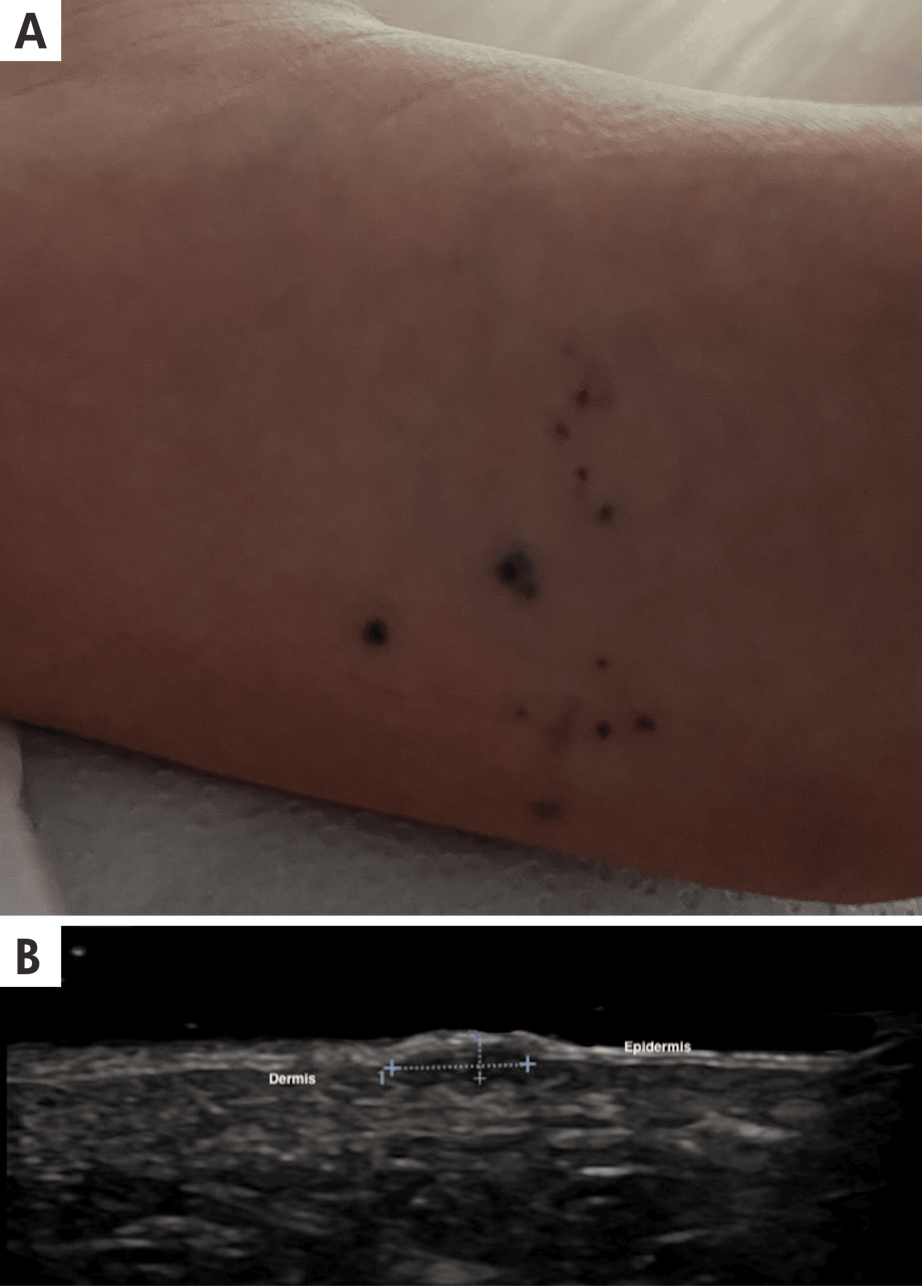
Dermatological ultrasound and clinical imaging of cherry angioma A: A 6-year-old male patient had maculopapular, cup-shaped, red-wine-colored lesions ranging from 1 mm to 4 mm, randomly distributed but grouped in the left plantar arch region. B: The longitudinal ultrasound view and grayscale ultrasound of the foot show between calipers (+) a small hypoechoic, exophytic nodule, causing upward displacement of the epidermis. This epidermis has the classic hyperechoic bilaminar appearance in the sole.

**Figure 5:**
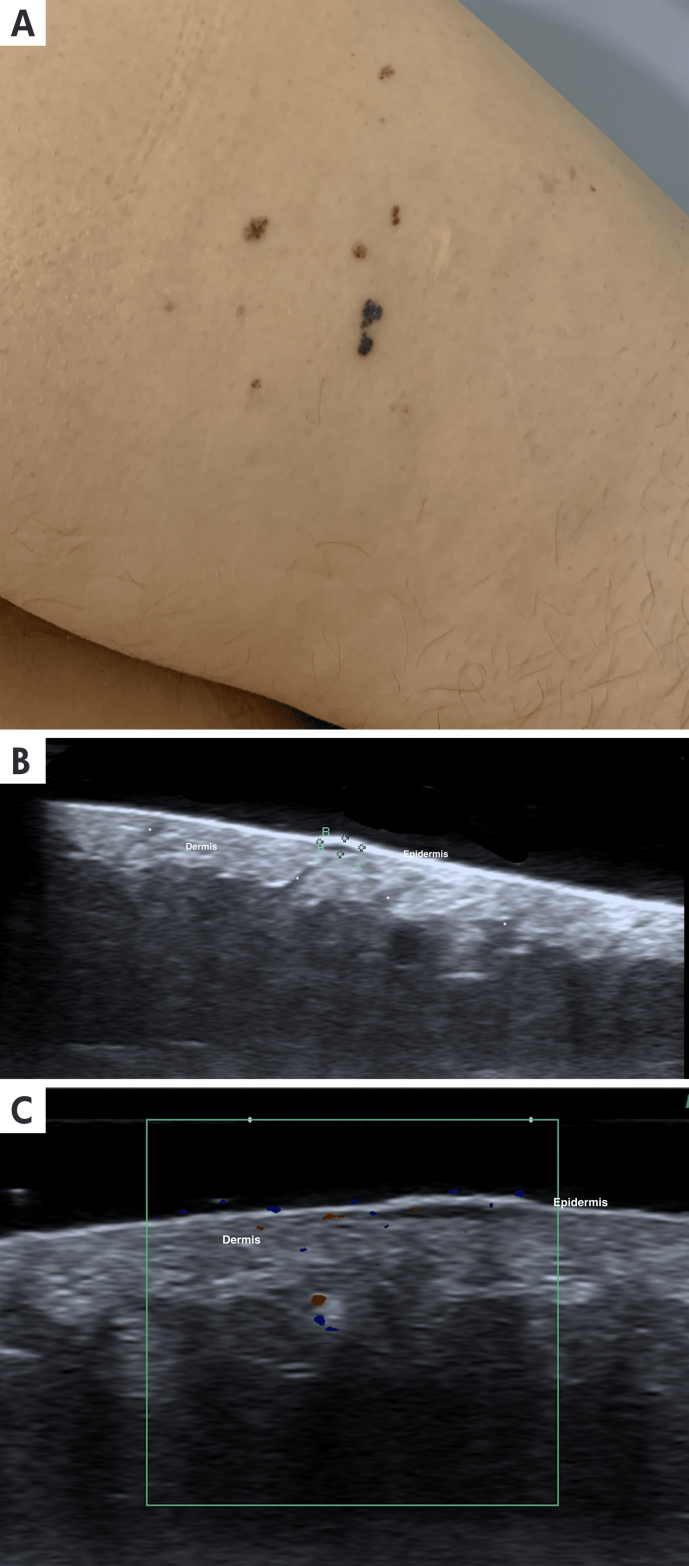
Dermatological ultrasound and clinical imaging of angiokeratoma A: A 46-year-old female patient with well-demarcated, keratotic, violaceous, and blackish papular lesions, ranging from 1 mm to 3 mm, located on the posterior aspect of the right thigh. B: The axial ultrasound view and grayscale ultrasound of the posterior aspect of the thigh show between the calipers (+) a small hypoechoic, exophytic nodule located in the dermo-epidermal junction, causing upward displacement of the epidermis. Asterisks (*) show the normal hair follicles. C. On Doppler, duplex color Doppler color ultrasound, there is a minimum increase in the vascularization on the periphery of the lesion.

**Figure 6:**
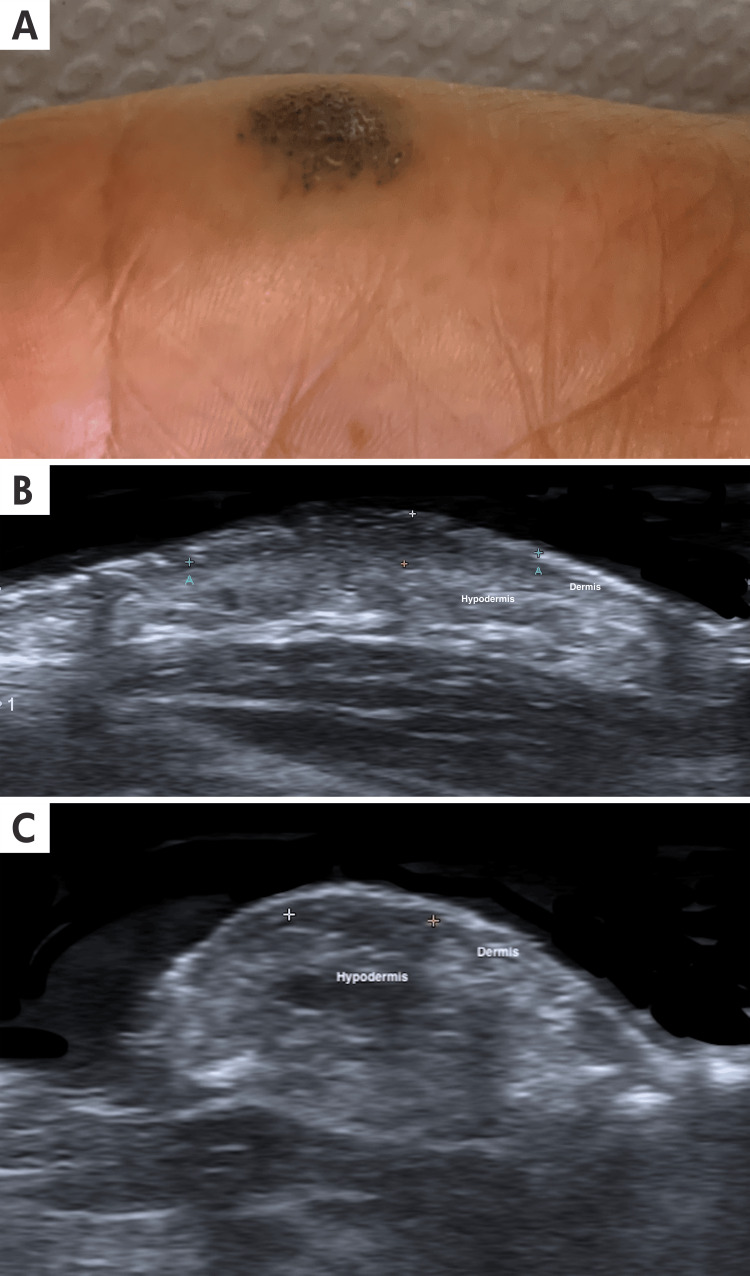
Dermatological ultrasound and clinical imaging of verrucous venous malformation A: A 51-year-old female patient with vascular dilations, papular in appearance, grouped to form a violaceous, rounded, irregular patch with a soft consistency and warty surface, approximately 1.5 cm in diameter, located in the left hypothenar region. B and C: The axial and longitudinal ultrasound view and grayscale ultrasound of the palmar aspect of the hypothenar left-hand show between calipers (+) a hypoechoic-like-band structure expanding the dermis and superficial hypodermis with poorly defined borders. There is some loss of definition in the dermo-hypodermis area.

Figures 1-6 illustrate key features such as anatomical location, morphological characteristics, and specific ultrasonographic patterns, complementing the textual descriptions and offering practical examples for accurate diagnosis and management.

## Discussion

Pyogenic granuloma

Clinical Findings

General information: Pyogenic granuloma, also referred to as lobular capillary hemangioma or telangiectasic granuloma, is not associated with granular tissue or pus. It refers to a common, acquired, benign vascular tumor characterized by rapid growth. It arises in tissues such as the skin and mucous membranes, particularly in sites of frequent trauma like the lips, gingiva, and fingers. It rarely occurs in other areas of the gastrointestinal tract [7,8].

The etiology of PG is still unknown, but it has been proposed that insults to the skin and mucous membranes result in an imbalance between pro- and anti-angiogenic factors, leading to the rapid growth of fragile and lobulated neovascular capillaries. Among periungual PG cases, it is reported that up to 30% occur in association with medications such as systemic and topical retinoids, antineoplastics, and various others. Other associations have been reported with chronic dermatoses. Similarly, the rare occurrence of disseminated PG has been documented in patients with severe nodulocystic acne using isotretinoin [8].

Clinical presentation: The typical presentation is a solitary, red, pedunculated papule that is very friable and exhibits rapid exophytic growth with a surface that can ulcerate. Bleeding from the lesion is a common finding and usually the initial symptom. It can also appear as a sessile plaque [8]. Some of these findings are shown in Figure 1.

Treatment: Pyogenic granuloma can be treated with surgical excision, curettage with cauterization, topical imiquimod 5% cream, neodymium-doped yttrium aluminum garnet (Nd:YAG) laser, cryosurgery, intralesional steroids, or flashlamp pulsed dye laser.

Ultrasound Findings

Pyogenic granuloma usually presents a well-defined, exophytic, or polypoid epidermal and dermal hypoechoic structure; it has smooth margins. The Doppler evaluation reveals hypervascularity within the lesion with arterial vessels, usually with high-speed velocity and visible pulsation, including in grayscale evaluation [4]. The detailed ultrasonographic characteristics of the lesion are illustrated in Figure 1.

Cutaneous angiosarcoma

Clinical Findings

General information: Cutaneous angiosarcoma is a malignant neoplasm arising from differentiated endothelial tissue. The incidence in the United States is reported at 0.5 new cases per 1,000,000 people a year. It is classified by the site of the primary tumor as cutaneous or visceral angiosarcoma. Both types can be further subclassified as primary or secondary, with secondary angiosarcomas often associated with prior ionizing radiation or chronic lymphedema. It is highly aggressive and primarily affects older patients, with 85% of cases occurring in individuals over 60 years of age, likely because of chronic sun exposure [9].

Clinical presentation: Cutaneous angiosarcoma can present with a diverse array of clinical manifestations, earning its reputation as a formidable mimicker. Initially, it may manifest as bruise-like lesions, violaceous nodules and plaques, and flat, infiltrating hemorrhagic areas. Cases have also been documented where lesions mimic conditions such as rosacea, xanthelasma, cellulitis, and facial angioedema. As the disease progresses, its presentation can resemble squamous cell carcinoma, basal cell carcinoma, malignant melanoma, and lymphomas. This broad spectrum of appearances complicates clinical diagnosis significantly. Clinicians must be aware that cAS is a rapidly spreading tumor capable of late-stage metastasis to lymph nodes or internal organs [10]. Some of these findings are shown in Figure 2.

Treatment: The five-year overall survival rate is estimated to be 33.5%. Initial treatment involves surgical excision, with adjunctive radiotherapy as necessary. If the tumor is unresectable or has metastasized, systemic therapy should be initiated, typically utilizing doxorubicin or paclitaxel [9].

Ultrasound Findings

Cutaneous angiosarcoma presents as a nodule or mass with mixed echogenicity, predominantly hypoechoic, with poorly defined borders. Usually, it affects the deep dermis and subcutaneous cellular tissue. On the periphery of the lesion and the non-nodular part of the tumor, mixed echogenicity is present. At the Doppler evaluation, usually irregular, tortuous, and multiple vascular structures are seen, with asymmetric neovascularization inside and on the periphery of the lesion. Arteriovenous shunts can be found [4]. The detailed ultrasonographic characteristics of the lesion are illustrated in Figure 2.

Extradigital glomus tumors

Clinical Findings

General information: Glomus tumors are small mesenchymal neoplasms originating from the neuromyoarterial glomus (glomus body), which functions as an arteriovenous shunt involved in thermoregulation within the skin. Glomus bodies are primarily found in the skin of the hands and feet, particularly in the nail beds and digital pads, as well as in the skin of the nose, ears, and along certain nerves and blood vessels. They are classified based on their location as either digital or extradigital, with the latter being less common. There are three histological types of GTs: solid GT, glomangioma, and glomangiomyoma, distinguished by cellular predominance and the presence or absence of a well-defined capsule. Although rare, the precise incidence of GTs remains unknown; they most commonly occur in subungual locations. Subungual presentations are twice as common in women as in men, whereas extradigital forms occur four times more frequently in men. While visceral GTs have been reported, they are extremely rare [11,12].

Clinical presentation and treatment: Glomus tumors typically appear in middle-aged adults as small, usually less than 0.5 cm, solitary nodules with reddish skin discoloration or bluish subungual spots. These lesions are known to cause excruciating pain upon light touch or exposure to cold. Generally, there are no associated neurological symptoms. Glomus tumors are commonly found in superficial soft tissues and generally exhibit a benign course, although occasional reports describe recurrent or malignant forms [11,12]. Some of these findings are shown in Figure 3. Surgical excision is possible since they are well-capsulated tumors.

Ultrasound Findings

Glomus tumors typically manifest as solid, hypoechoic nodules with well-defined, regular borders on ultrasound, usually with small hypoechoic, serpentine tubular structures within that correspond to a vascular vessel; the pulsation of this vessel can sometimes be visible even at the grayscale ultrasound evaluation. Doppler ultrasound confirms significant vascularity within the tumor; the velocity in this vessel is usually higher than that of the normal vessel located in the anatomical area where the tumor is located. They are usually located in the superficial dermis without the involvement of deeper layers, even if they can have large sizes. [11,12] The detailed ultrasonographic characteristics of the lesion are illustrated in Figure 3.

Cherry angiomas

Clinical Findings

General information: Cherry angiomas, also known as Campbell de Morgan spots, are benign vascular proliferations of endothelial cells. These lesions are usually asymptomatic and are often associated with aging [13,14].

Clinical presentation: They present as small, bright red to purple papules, commonly appearing on the trunk and extremities. The diagnosis is primarily clinical, but dermatoscopy can aid in visualization, showing red, purple, or blue-black lacunae. Histopathological examination, if performed, reveals dilated capillary loops lined by flattened endothelial cells [14]. Some of these findings are shown in Figure 4.

Treatment: While treatment is generally not necessary unless for cosmetic reasons or symptomatic lesions, several modalities are available. Pulsed dye laser (PDL) is preferred because of its efficacy and lower pain levels compared to potassium-titanyl-phosphate (KTP) and electrodesiccation (ED). The Nd:YAG laser is also effective, particularly in darker-skinned individuals, to minimize pigmentary changes. Cherry angiomas have an excellent prognosis. There is no known malignant potential, and they rarely cause significant morbidity [13,14].

Ultrasound Findings

Ultrasound imaging of ChA revealed thin, hypoechoic, and superficial lesions confined to the upper dermis with well-defined borders, causing upward displacement of the epidermis. Doppler analysis did not detect any vascular flow within these lesions due to the low flow of the capillaries inside them. Usually, ultrasound evaluation is not necessary because the diagnosis is clinical, but in younger patients or atypical locations, ultrasound evaluation can exclude other diagnoses and confirm the presence of ChA [15]. The detailed ultrasonographic characteristics of the lesion are illustrated in Figure 4. 

Angiokeratoma

Clinical Findings

General information: Angiokeratomas are benign vascular ectasias of the superficial dermal blood vessels, characterized by overlying hyperkeratosis or acanthosis of the epidermis. They can develop due to increased venous pressure, as seen in conditions such as pregnancy. Clinically, five types of Ak have been described: angiokeratoma corporis diffusum, finger angiokeratoma, angiokeratoma of the scrotum or vulva, circumscribed naeviform angiokeratoma, and the most common type, solitary or multiple angiokeratomas [16,17].

Clinical presentation and treatment: Angiokeratomas typically appear as multiple keratotic papules that range in size from 2 mm to 10 mm. These papules have a dark red to black color. The surface is rough or warty due to hyperplasia of the stratum corneum. The lesions can be flat or slightly elevated and are generally asymptomatic but may bleed, thrombose, or become traumatized. Initially, they often appear as painless, non-keratotic papules or plaques, which may later develop hyperkeratosis and become painful due to the lesion's natural progression or local pressure [16,17]. Some of these findings are shown in Figure 5. Angiokeratomas do not possess malignant potential. Therapeutic approaches typically include excision, electrodesiccation, cryotherapy, or laser ablation [16,17].

Ultrasound Findings

On ultrasound, Ak can be identified as band-like epidermal and dermal structures with thickening, undulation, and irregularities of the epidermis. In the surrounding tissue, thickening and decreased echogenicity can be found. At the Doppler evaluation, these lesions show hypovascularity. The detailed ultrasonographic characteristics of the lesion are illustrated in Figure 5.

Verrucous venous malformation

Clinical Findings

General information: Verrucous venous malformation, previously called 'verrucous hemangioma,' is an uncommon congenital simple vascular malformation that may appear at birth or develop later in adulthood. They are predominantly found on the lower extremities, with a unilateral presentation in roughly 95% of cases [18].

Clinical presentation: They typically present as warty, bluish vascular papules, plaques, or nodules. They are characterized by reactive epidermal acanthosis, papillomatosis, hyperkeratosis, and extension into the subcutaneous tissues. Lesions tend to enlarge and become increasingly keratotic in response to injury, infection, or inadequate treatment. The diagnosis of VVM is primarily clinical, supported by histopathological examination [19]. Some of these findings are shown in Figure 6.

Treatment: The preferred treatment for VVM is surgical excision, often combined with laser therapy. This approach is recommended because the crucial pathologic changes are concentrated in the subcutaneous tissue. Superficial ablative therapies (laser, cryosurgery, and electrocautery) are generally followed by recurrence and are not recommended as standalone treatments [19]. The prognosis for VVM is good with appropriate treatment. Surgical excision has shown favorable outcomes, with a low recurrence rate when the lesion is completely removed. Long-term follow-up is recommended to monitor for potential recurrences. [19]

Ultrasound Findings

On ultrasound, VVM lesions usually show variable degrees of thickening, irregularities, and undulation of the epidermis. These lesions infiltrate the dermis with thickening and decrease echogenicity of the dermis with ill-denied hyperechoic hypodermis. Most of these lesions demonstrate subcutaneous fat infiltration. Doppler evaluation often reveals low vascular density or an absence of signal within the lesion, signifying the low-flow or static nature of the blood within these malformed venous channels. A venous spectrum is commonly observed when analyzing these lesions. There is a notable loss of the usual definition between the dermis and epidermis, indicative of tissue disruption and remodeling. Hyperechoic channels, representing abnormal venous structures typical of VVM, can be observed within the dermis and extend into the subcutaneous tissue [20]. The detailed ultrasonographic characteristics of the lesion are illustrated in Figure 6.

Limitations of the study

This study has several limitations. First, the retrospective design and random selection of cases may introduce selection bias, potentially affecting the generalizability of the findings and not fully representing the spectrum of VA. The small sample size and focus on less common anomalies further limit the ability to make broader conclusions about dermatological ultrasound’s effectiveness across all VA. Additionally, the requirement for histological confirmation and complete clinical records may have excluded cases with less thorough documentation, impacting sample representativeness. Finally, the lack of long-term follow-up data restricts the assessment of clinical outcomes and recurrence. Future research should address these limitations by including larger, more diverse populations and extending follow-up periods.

## Conclusions

Dermatological ultrasound proves essential in diagnosing and managing less common vascular anomalies. This study demonstrates its effectiveness in distinguishing between various vascular anomalies, such as PG, cAS, extradigital GT, ChA, Ak, and VVM. The modality’s ability to provide detailed imaging helps in accurate diagnosis and effective treatment planning, reinforcing its value in clinical practice for vascular anomaly management. Future studies should build on these findings to further validate and refine diagnostic approaches.
